# Supplementary material for: Cytotoxicity of Deoxynivalenol after Being Exposed to Gaseous Ozone
Source: Toxins (Basel). 2019 Nov 2;11(11):639. doi: 10.3390/toxins11110639 (PMC6891369; doi:10.3390/toxins11110639)
Supplement: Supplementary file 1 [file toxins-11-00639-s001.pdf]

Supplementary Materials: Cytotoxicity of Deoxynivalenol after Being Exposed to Gaseous Ozone

Dongliang Ren, Enjie Diao, Hanxue Hou, Peng Xie, Ruifeng Mao, Haizhou Dong and Shiquan Qian

Density Observation of HepG2 and Hela Cells

The density observation of human hepatic carcinoma (HepG2) and Henrietta Lacks (Hela) cells further verified the changes of cell viability for HepG2 and Hela cells (Figures 1 and 2). The normal HepG2 and Hela cells are fusiform or polygon, and grow adhered to the wall. They are evenly distributed in the wall and in close contact. The densities and numbers of HepG2 and Hela cells exposed to deoxynivalenol (DON) without ozone treatment were significantly reduced (Figures 1B and 2B). With the increase of the ozone exposure time, the densities and numbers of the two kinds of cells were evidently improved. In addition, there were no significant differences between the negative control and that exposed to ozone for 15 min.

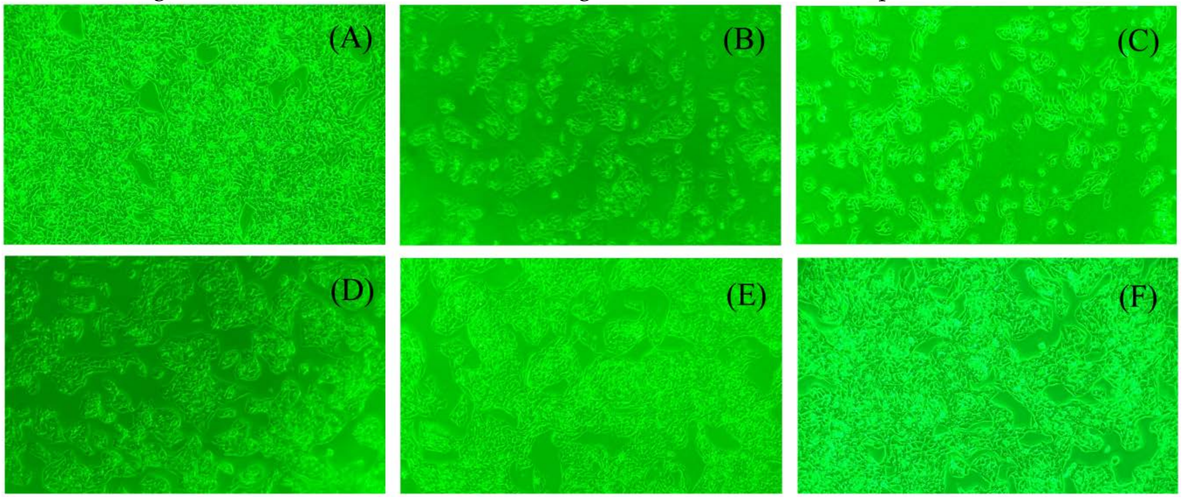

Figure S1. Density changes of human hepatic carcinoma (HepG2) cells exposed to deoxynivalenol (DON) solution treated by ozone for different times: (**A**) control without DON, (**B**) 0 min, (**C**) 5 min, (**D**) 10 min, (**E**) 15 min, and (**F**) 20 min.

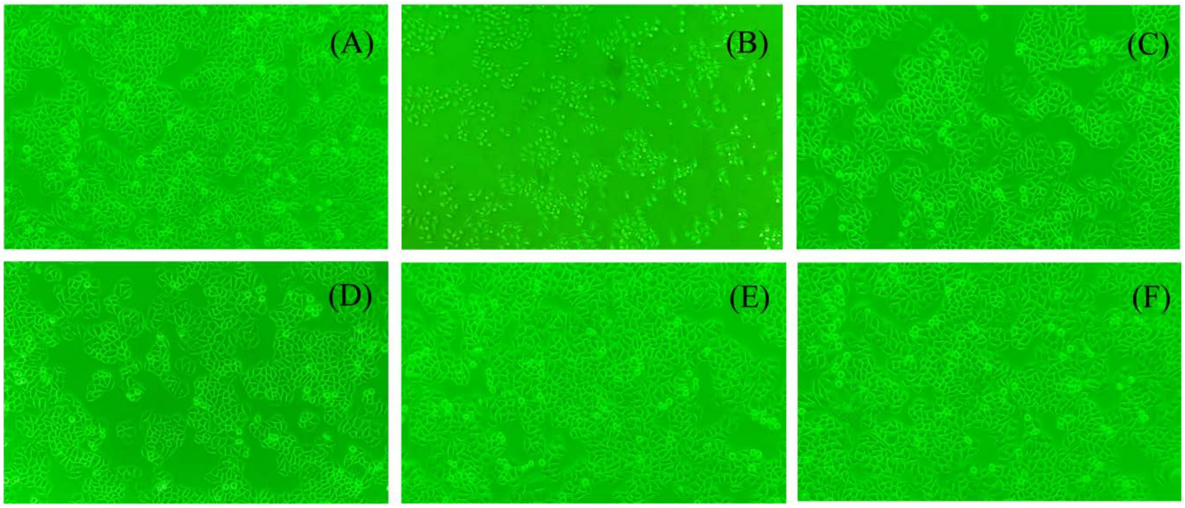

Figure S2. Density changes of Henrietta Lacks (Hela) cells exposed to DON solution treated by ozone for different times: (**A**) control without DON, (**B**) 0 min, (**C**) 5 min, (**D**) 10 min, (**E**) 15 min, and (**F**) 20 min.
